# Trends of atherosclerosis-related mortality in adults with diabetes: A cross-sectional analysis of U.S. national data

**DOI:** 10.1016/j.athplu.2025.04.001

**Published:** 2025-05-02

**Authors:** Yusuf Hasan Ali, Fakhar Latif, Fatimah Hoda, Azeem Hassan, Huda Ahmed, Raheel Ahmed, Vikash Jaiswal

**Affiliations:** a Department of Internal Medicine, Dow University of Health Sciences, Karachi, Pakistan; b National Heart & Lung Institute, Imperial College London, London, UK; c Department of Cardiology, Royal Brompton Hospital, London, UK; d Department of Cardiovascular Research, Larkin Community Hospital, South Miami, FL, USA

**Keywords:** Atherosclerosis, Diabetes mellitus, Mortality, United States, Centre for Disease control, WONDER database

## Abstract

**Background:**

Diabetes mellitus is a rapidly growing global health issue, projected to affect 643 million adults by 2030. Atherosclerosis, a prevalent complication of diabetes, significantly contributes to morbidity and mortality among affected individuals. This study aimed to analyze mortality trends associated with atherosclerosis in diabetic patients aged ≥45 years, with particular focus on variations by sex, race, urban-rural classification, and geographical regions in the US from 1999 to 2020.

**Methods:**

We conducted a study using data from the CDC WONDER database, identifying atherosclerosis-related deaths in diabetic patients. We calculated age-adjusted mortality rates (AAMRs) per 100,000 population and analyzed trends over time using Joinpoint regression to assess annual percentage changes (APC) and average annual percentage changes (AAPC).

**Results:**

A total of 674,582 atherosclerosis-related deaths were recorded in diabetic patients from 1999 to 2020, with a higher prevalence in men (57.40 %). The majority of deaths occurred in NH White individuals (81.70 %). Overall, AAMRs declined from 32.8 in 1999 to 25.8 in 2020. A significant decrease was observed from 1999 to 2014 (APC: −2.61, p < 0.05), followed by stability (2014–2018) and a subsequent rise (APC: 6.97, p < 0.05) till 2020. Sex-stratified analysis indicated persistently higher AAMRs in men, with a significant increase from 2018 to 2020 (APC: 7.33, p < 0.05). Racial disparities were evident, with NH Black individuals demonstrating the highest AAMRs. Geographic analysis revealed higher AAMRs in nonmetropolitan areas, with notable state-level variations. All census regions exhibited an initial decline, followed by a significant rise in AAMRs post-2018 (p < 0.05).

**Conclusion:**

Despite initial declines, recent trends indicate a resurgence in atherosclerosis-related mortality among diabetic patients, particularly in specific racial groups, rural areas, and certain regions. These findings underscore the need for targeted interventions to address disparities and improve cardiovascular outcomes in diabetic populations.

## Highlights

• Atherosclerosis-related mortality in diabetic patients aged ≥45 initially declined but saw a notable resurgence from 2018 to 2020, particularly among specific racial groups, rural areas, and the Southern region of the U.S.
• Men, Non-Hispanic Black, and American Indian populations exhibited higher age-adjusted mortality rates (AAMRs) than other demographic groups, with significant disparities evident across race and geographical classifications.
• Findings emphasize the critical need for targeted interventions to address cardiovascular health disparities and improve outcomes among diabetic patients, especially in rural areas and high-risk racial groups.

## 1 Introduction

Diabetes mellitus is an escalating global health concern. According to a report by the International Diabetes Federation, it is projected that 643 million adults will be affected by diabetes by 2030 [1]. Diabetes mellitus, characterized by chronic hyperglycemia, arises from insufficient insulin production, impaired insulin action, or a combination of both [2]. Recent studies demonstrate the growing threat of diabetes mellitus, ranking it as the third most prevalent non-communicable disease worldwide, following cardiovascular diseases (CVD) and cancer [3]. One of the major complications of diabetes mellitus is atherosclerosis. Most of the literature on the onset of diabetes complications is largely associated with atherosclerosis suggesting that chronic hyperglycemia disrupts vascular homeostasis causing endothelial dysfunction thus beginning the process of pathological events that lead to atherosclerosis [4]. As atherosclerosis progresses, it forms plaque in the intimal layer of the arterial wall, and the subsequent rupture of the plaque causes thrombogenesis. Narrowing of the lumen of blood vessels and thrombotic events often lead to fatal consequences [2]. Furthermore, insulin resistance upregulates vascular NADPH oxidase, increasing reactive oxygen species (ROS) production that oxidizes LDL particles to atherogenic ox-LDL [5]. Dyslipidemia characteristic of diabetes (elevated triglycerides, sdLDL) activates the NLRP3 inflammasome via TLR4/NF-κB signaling, triggering IL-1β release and endothelial junction disruption [6]. Advanced glycation end products (AGEs) crosslink vascular collagen through RAGE receptors, reducing arterial elasticity while activating PKC-β to inhibit eNOS-derived nitric oxide, further contributing to the development of atherosclerosis in diabetes [7].

To mitigate the public health impact of diabetes complications, early diagnosis of diabetes and prediabetes is essential. Utilizing blood sugar testing, hemoglobin A1_c_ and OGTT criteria for each type of diabetes allows for detection at its early stages. Effective management hinges on timely diagnosis and maintaining blood sugar control throughout the patient's life under physician guidance and timely use of appropriate medicines. It is also crucial to educate patients on managing their blood sugar levels through regular exercise, adopting a healthy lifestyle, dietary modifications, weight loss, avoiding smoking, and cholesterol and blood pressure control. Even a few months of uncontrolled hyperglycemia can significantly increase the risk of complications. In the past two decades, the introduction of new drug classes, such as SGLT-2 inhibitors and GLP-1 analogs, has greatly improved diabetes care [8]. These medications not only help control blood sugar but also reduce the risk of cardiovascular disease. Timely use of medications, including insulin when needed, is essential for keeping blood sugar levels under control and delaying or preventing complications [9]. By implementing these strategies, the burden of diabetes complications can be significantly reduced. Given the association between diabetes and development of atherosclerotic disease, it is of paramount importance to identify mortality trends and disparities in a cross-sectional study and this study aims to fill this void.

Atherosclerosis remains a leading cause of mortality in diabetic adults, yet prior studies primarily assess trends in the general population, potentially overlooking disparities in this high-risk group. While current literature show declining ischemic heart disease mortality, it is unclear whether similar reductions have occurred in diabetic individuals [10]. Therefore, this study aimed to analyze mortality trends associated with atherosclerosis in diabetic individuals aged ≥45 years using nationally representative CDC WONDER data. Age-adjusted mortality rates (AAMRs) were computed per 100,000 total U.S. residents, to ensure comparability with national mortality statistics and facilitate direct public health and policy discussions.

## 2 Methods

### 2.1 Data sources

Our analysis involved the data from the Centers for Disease Control and Prevention, Wide-Ranging Online Data for Epidemiologic Research (CDC WONDER) database [11]. Multiple Cause-of-Death Public Use Record death certificates were used to analyze deaths in individuals ≥45 years of age in which atherosclerosis was mentioned as either contributing or underlying cause of death, while diabetes mellitus was mentioned as the multiple causes of death. We used the following International Classification of the Diseases, Tenth Revision (ICD-10) codes to identify atherosclerosis and diabetes mellitus-related cases: I25.0, I25.1, I67.2, I70.0, I70.1, I70.2, I70.8, I70.9, and E10-E14.

### 2.2 Ethics committee

Since the publicly available, anonymized government data was used, the institutional review board approval was not required. The study was also conducted per the STROBE (Strengthening the Reporting of Observational Studies in Epidemiology) guidelines [12].

### 2.3 Data extraction

The number of atherosclerosis and diabetes mellitus-related deaths and population size were extracted from 1999 to 2020. The data on age, sex, race and ethnicity, region, and state were also abstracted. We then selected patients aged ≥45 years and divided them into 10-year age groups. Thereafter, we categorized patients based on sex and studied individual mortality trends for both sexes. For race, patients were stratified into White, Black, Hispanic, Asian/Pacific Islander, and American Indian/Alaskan Native. The population was further stratified into metropolitan and non-metropolitan areas using the National Center for Health Statistics Urban-Rural Classification Scheme. Regions were categorized into Northeast, Midwest, South, and West, following the Census Bureau-defined regional divisions [13].

### 2.4 Statistical analysis

The number of atherosclerosis and diabetes mellitus-related deaths was divided by the total corresponding population to calculate the crude death rates for individual years. The annual mortality rates were calculated per 100,000 total U.S residents, and their corresponding 95 % confidence intervals (CIs) were also determined. The mortality rates were adjusted for age by standardizing to the year 2000 US standard population [14]. Temporal trends in mortality were examined to deduce changes in slope using Joinpoint Regression Program version 4.7.0.0, which models consecutive linear segments on a log scale connected by Joinpoints, where the segments converge [15]. Annual percentage changes (APC) and Average Annual Percentage Changes (AAPCs) were calculated, with their corresponding 95 % CIs for the line segments linking a Joinpoint. Slopes were considered increasing or decreasing if the estimated slope differed significantly from zero. The statistical significance was determined by 2-sided t-testing (P = 0.05).

## 3 Results

Between 1999 and 2020, a total of 674,582 atherosclerosis-related deaths were recorded among diabetic patients. Atherosclerosis-related mortality was higher in men at 57.40 % than in women at 42.60 %. Regarding racial distribution, the majority of deaths occurred in NH White individuals at 81.70 %, followed by NH Black at 14.40 %, Hispanic/Latino at 7.40 %, NH Asian/Pacific Islander at 3.10 %, and NH American Indian/Alaska Native at 0.80 %. [Supplementary Table 1]. Information regarding the location of death was available for 674,582 of these deaths. Among these, 38.00 % occurred in medical facilities, 35.40 % at home, 20.40 % in nursing homes or long-term care facilities, 4.30 % in other places, 0.05 % in unknown places, and 0.04 % in hospices [Supplementary Table 2].

### 3.1 Annual trend for atherosclerosis-related AAMR

**Fig. 1:**
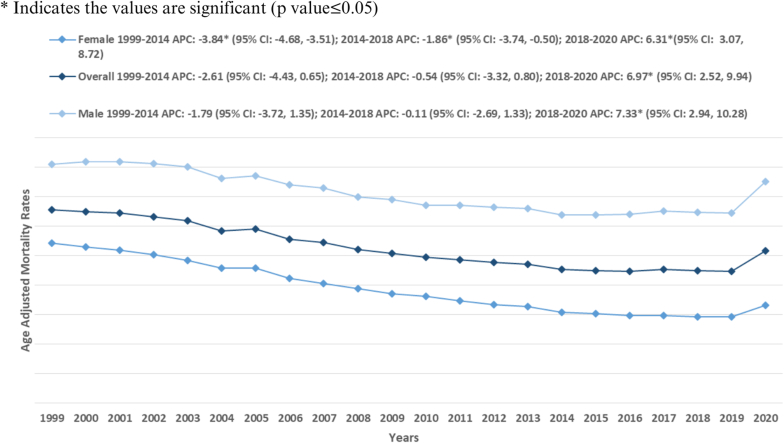
Atherosclerosis-related mortality trends in adults with diabetes stratified for gender in 1999–2020.

The AAMRs for atherosclerosis-related mortality in diabetic patients have gradually declined from 32.8 in 1999 to 25.8 in 2020. Additionally, a noticeable decrease in AAMR was seen from 1999 to 2014 [APC: −2.61 (−4.43, 0.65)]. Subsequently, the AAMRs showed a period of stability from 2014 to 2018 [APC: −0.54 (−3.32, 0.80)]. Finally, the AAMRs increased significantly till 2020 [APC: 6.97∗ (2.52, 9.94)] [Fig. 1, Table 1, Supplementary Table 3].

**Table 1:** Deaths, overall AAMRs, and annual percent change (APC) of atherosclerosis-related mortality in diabetic patients in the United States, 1999 to 2020.

Variable	Deaths (n)	AAMR (95 % CI)	Trend Segment	Year Interval	APC (95 % CI)	AAPC (95 % CI)
Overall	674,582	26.0 (26.0, 26.1)	1	**1999**–**2014**	−2.61 (−4.43, 0.65)	−1.34^a^ (−1.64, −1.06)
			2	**2014**–**2018**	−0.54 (−3.32, 0.80)	
			3	**2018**–**2020**	6.97^a^ (2.52, 9.94)	
Sexual Disparities						
Females	287,542	19.0 (19.0, 19.1)	1	**1999**–**2014**	−3.84^a^ (−4.68, −3.51)	−2.53^a^ (−2.75, −2.38)
			2	**2014**–**2018**	−1.86^a^ (−3.74, −0.50)	
			3	**2018**–**2020**	6.31^a^ (3.07, 8.72)	
Males	387,040	35.3 (35.2, 35.4)	1	**1999**–**2014**	−1.79 (−3.72, 1.35)	−0.64^a^ (−0.94, −0.36)
			2	**2014**–**2018**	−0.11 (−2.69, 1.33)	
			3	**2018**–**2020**	7.33^a^ (2.94, 10.28)	
Racial Disparities						
White	504,108	24.5 (24.4, 24.5)	1	**1999**–**2014**	−2.53 (−3.46, −2.03)	−1.35^a^ (−1.57, −1.18)
			2	**2014**–**2018**	−0.29 (−2.49, 1.02)	
			3	**2018**–**2020**	5.60 (2.48, 7.72)	
Black or African American	88,845	37.6 (37.3, 37.8)	1	**1999**–**2018**	−2.89 (−3.43, −2.45)	−1.38^a^ (−1.99, −0.99)
			2	**2018**–**2020**	14.21 (4.26, 19.16)	
American Indian or Alaska Native	5076	36.1 (35.0, 37.1)	1	**1999**–**2020**	−0.68 (−1.12, −0.14)	−0.68 (−1.12, −0.14)
Hispanic or Latino	55,159	29.3 (29.1, 29.6)	1	**1999**–**2018**	−3.37 (−3.78, −3.00)	−2.04^a^ (−2.49, −1.70)
			2	**2018**–**2020**	11.59 (4.52–15.77)	
Asian or Pacific Islander	19,052	20.0 (19.7, 20.3)	1	**1999**–**2018**	−2.99 (−3.47, −2.56)	−1.67^a^ (−2.15, −1.29)
			2	**2018**–**2020**	11.82 (4.03, 16.08)	
Rural/Urban Disparities						
Nonmetropolitan areas	121,755	26.6 (26.5, 26.8)	1	**1999**–**2001**	0.58 (−1.74, 2.72)	−0.55^a^ (−0.73, −0.37)
			2	**2001**–**2010**	−2.47 (−4.10, −2.12)	
			3	**2010**–**2018**	−0.56 (−1.09, - 0.14)	
			4	**2018**–**2020**	7.36 (5.04, 8.85)	
Metropolitan area	552,827	25.9 (25.8, 26.0)	1	**1999**–**2014**	−2.76 (−5.12, 1.61)	−1.47^a^ (−1.83, −1.11)
			2	**2014**–**2018**	−0.78 (−4.11, 0.90)	
			3	**2018**–**2020**	7.17 (2.27, 10.43)	
Regional Disparities						
North-eastern Census Region	134,166	26.3 (26.2, 26.5)	1	**1999**–**2018**	−2.84^a^ (−3.14, −2.57)	−1.34^a^ (−1.72, −1.08)
			2	**2018**–**2020**	14.12 (7.60, 17.58)	
Mid-western Census Region	159,376	27.3 (27.1, 27.4)	1	**1999**–**2014**	−2.39 (−3.73, −0.64)	−1.26^a^ (−1.53, −1.08)
			2	**2014**–**2018**	−0.65 (−3.27, 0.56)	
			3	**2018**–**2020**	6.34 (2.15, 9.06)	
Southern Census Region	221,331	23.4 (23.3, 23.5)	1	**1999**–**2011**	−2.54 (−4.69, −1.89)	−1.01^a^ (−1.39, −0.77)
			2	**2011**–**2018**	−0.65 (−2.25, 1.07)	
			3	**2018**–**2020**	7.25 (1.79, 10.26)	
Western Census Region	159,315	28.8 (28.6, 28.9)	1	**1999**–**2016**	−2.89 (−3.35, −2.56)	−1.90^a^ (−2.29, −1.63)
			2	**2016**–**2020**	2.40 (−0.46, 8.21)	

† AAMR: Age-adjusted Mortality Rates; APC: Annual Percent Change; AAPC: Average Annual Percentage Change.

a Indicates the values are significant (p value= >0.05).

### 3.2 Atherosclerosis-related AAMR stratified by sex

Throughout the study duration, men consistently demonstrated higher AAMRs than females, despite both groups showing a downward trend (overall AAMR Males: 35.3 [95 % CI: 35.2, 35.4]; Females: 19.0 [95 % CI: 19.0, 19.1]) [Table 1]. The AAMRs for males between 1999 and 2014 showed a decreasing trend [APC: −1.79∗ (−3.72, 1.35)]. There was a further decrease from 2014 to 2018 [APC: −0.11 (−2.69, 1.33)]. From 2018 to 2020, the AAMRs increased steadily [APC: 7.33∗ (2.94, 10.28)].

For females, a similar pattern was observed. The AAMRs decreased steadily from 1999 to 2014 [APC: −3.84∗ (−4.68, −3.51)], followed by a further decrease from 2014 to 2018 [APC: −1.86∗ (−3.74, −0.50)]. Finally, the AAMRs increased till 2020 [APC: 6.31∗ (3.07, 8.72)] [Fig. 1, Table 1, Supplementary Table 4].

### 3.3 Atherosclerosis-related AAMR stratified by race

**Fig. 2:**
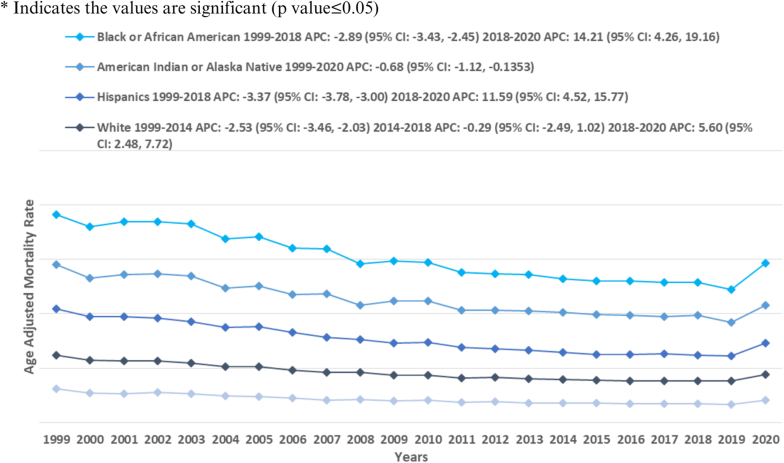
Atherosclerosis-related mortality trends in adults with diabetes stratified for differential racial groups in 1999–2020.

When stratified by race/ethnicity, NH Blacks had the highest AAMR, followed by NH American Indians, Hispanics, NH Whites and Asian or Pacific Islander (overall AAMR NH White: 24.5 [95 % CI: 24.4, 24.5]; NH Black or African American: 37.6 [95 % CI: 37.3, 37.8]; NH American Indian or Alaska Native: 36.1 [95 % CI: 35.0, 37.1]; Hispanic or Latino: 29.3 [95 % CI: 29.1, 29.6]; NH Asian or Pacific Islander: 20.0 [95 % CI: 19.7, 20.3]) [Table 1]. Asian/Pacific Islander and Hispanic/Latino showed a gradual decline in mortality rate from 1999 till 2018 [APC: Asian or Pacific Islanders: −2.99∗ (−3.47, −2.56); Hispanic/Latino: −3.37∗ (−3.78, −3.00)], followed by a much sharper rise for till 2020 [APC: Asian or Pacific Islander: 11.82∗ (4.03, 16.08); Hispanic/Latino: 11.59∗ (4.52, 15.77)]. Conversely, NH Whites showed a slight decline from 1999 to 2014 [APC: −2.53∗ (−3.46, −2.03)], which was followed by a period of stability from 2014 to 2018 [APC: −0.29 (−2.49, −1.02)]. Subsequently, a sharp rise was seen in the AAMRs till 2020 [APC: 5.60∗ (2.49, 7.72)]. American Indian/Alaska Native remains an exception, with stabilized AAMRs throughout the study period [APC: −0.68 (−1.12, −0.14)]. AAMRs observed for NH Black/African American, showed a decline from 1999 to 2018 [APC: −2.89 (−3.43, −2.45)], followed a sharp rise till 2020 [APC: 14.21∗ (4.26, 19.16)] [Fig. 2, Table 1, Supplementary Table 5].

### 3.4 Atherosclerosis-related AAMR stratified by geography

**Fig. 3:**
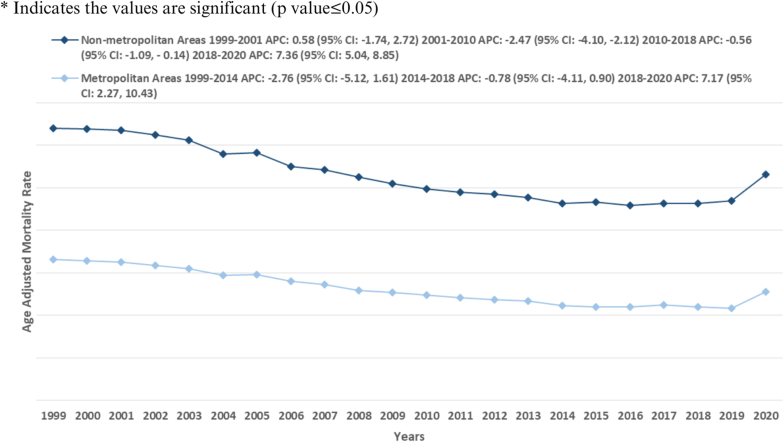
Atherosclerosis-related mortality trends in adults with diabetes stratified for metropolitan vs. non-metropolitan in 1999–2020.

***Rural-Urban Classification:*** Similarly, nonmetropolitan areas showed greater AAMRs for mortality due to atherosclerosis than metropolitan areas, with overall AAMRs of 26.6 (95 % CI: 26.5, 26.8) and 25.9 (95 % CI: 25.8, 26.0), respectively [Table 1]. Non-metropolitan areas demonstrated a period of stability from 1999 to 2001 [APC: 0.58 (−1.74, −0.71)]. This was followed by a period of another significant reduction until 2010 [APC: −2.47∗ (−4.10, −2.12)]. AAMRs remained stable from 2010 to 2018 [APC: −0.56 (−1.09, 0.14)], followed by an increase till 2020 [APC: 7.36∗ (5.04, 8.85)]. Furthermore, the AAMRs of metropolitan areas significantly decreased from 1999 to 2014 [APC: −2.76 (−5.12, 1.61)], followed by another decrease from 2014 to 2018 [APC: −0.78 (−4.11, 0.90)]. Subsequently, the AAMRs increased significantly from 2018 to 2020 [APC: 7.17∗ (2.27, 10.43)] [Fig. 3, Table 1, Supplementary Table 6].

***States:*** Additionally, there was a notable disparity in AAMR across the states, with Utah having the lowest AAMR of 15.0 (95 % CI: 14.4, 15.6) and District of Columbia having the highest AAMR of 53.1 (95 % CI: 51.0, 55.3). It is important to acknowledge that the states ranked in the top 90th percentile for AAMRs, including California, Ohio, West Virginia, Oklahoma, Vermont, and District of Columbia, had AAMRs almost thrice as much as the AAMRs of states that fell into the bottom 10th percentile, such as Utah, Alabama, Arkansas, Massachusetts, Florida, and Idaho. [Supplementary Fig. 1, Supplementary Table 7].

***Census Regions:*** Among the four census regions, the Western region consistently exhibited the highest AAMRs throughout the study duration, followed sequentially by the Midwestern, Northeastern, and Southern regions (overall AAMR Northeast: 26.3 [95 % CI: 26.2, 26.5]; Midwest: 27.3 [95 % CI: 27.1, 27.4]; South: 23.4 [95 % CI: 23.3, 23.5]; West: 28.8 [95 % CI: 28.6, 28.9]). A similar trend of atherosclerosis-related AAMRs was observed across the four census regions, with a marked decline, followed by a significant increase. In the Northeastern region, the AAMR substantially decreased from 1999 to 2018 [APC: −2.84∗ (−3.14, −2.57)], before experiencing a prominent rise from 2018 to 2020 [APC: 14.12∗ (7.60, 17.58)]. Meanwhile, the AAMR for the Midwest steeply reduced from 1999 to 2014 [APC: −2.39∗ (−3.73, −0.64)], followed by a less dramatic reduction from 2014 to 2018 [APC: −0.65 (−3.27, 0.56)], and subsequently, a drastic escalation from 2018 to 2020 [APC: 6.34∗ (2.15, 9.06)]. Similarly, the Southern regions also demonstrated an downward trend of atherosclerosis-related AAMRs between 1999 and 2011 [APC: −2.54 (−4.69, −1.89)]. After this, the AAMRs demonstrated stability from 2011 to 2018 [APC: −0.65 (−2.25, 1.07)], followed by another increase in AAMRs till 2020 [APC: 7.25∗ (1.79, 10.26)]. Finally, the Western regions first showed decline in the AAMR from 1999 to 2016 [APC: −2.89∗ (−3.35, −2.56)], which was followed by an increase till 2020 [APC: 2.39 (−0.46, 8.21)] [Supplementary Fig. 2, Table 1, Supplementary Table 8].

## 4 Discussion

The current study is a 20-year analysis of mortality data from the Centers for Disease Control and Prevention, in which we report several key findings. First, there was an overall decline in mortality rates from 1999 to 2018, followed by a significant rise till 2020. Second, men were found to have a higher AAMR than women. Third NH Blacks had a significantly higher AAMR than other racial groups. Nonmetropolitan areas were associated with greater AAMRs for mortality due to atherosclerosis as was the western region when compared with other census regions. These results, which pertain to the overall population studied, represent important implications in regard to disease management guidelines and health policy.

The potential reasons for higher rates of mortality from 2018 to 2020 are multifactorial. Infection by COVID-19 can be directly and indirectly associated with exacerbating cardiovascular disease as it is associated with an increase in thromboembolic events [16,17]. COVID-19 induces a hypercoagulable state characterized by elevated D-dimer levels, platelet activation, and increased tissue factor expression, further predisposing these patients to myocardial infarction, stroke, and venous thromboembolism [18]. Myocardial injury is also exacerbated through direct viral effects, cytokine-induced myocardial dysfunction, and an imbalance between oxygen supply and demand [19]. Diabetes further compounds these risks by promoting insulin resistance, hyperglycemia, and an impaired immune response, all of which contribute to prolonged viral replication, increased inflammation, and susceptibility to secondary infections [20]. As a result, patients with both diabetes and atherosclerosis experience a more severe disease course, higher rates of cardiovascular complications, and increased mortality, underscoring the need for vigilant thromboprophylaxis, glycemic control, and cardiac monitoring in this high-risk population. Importantly, certain antidiabetic drugs may influence the cardiovascular risk profile in this population. SGLT-2 inhibitors, such as empagliflozin, dapagliflozin, provide cardiovascular benefits by reducing heart failure risk, improving endothelial function, and exerting anti-inflammatory effects [21]. Similarly, GLP-1 receptor agonists, such as liraglutide, semaglutide, have anti-atherosclerotic and anti-inflammatory properties, potentially reducing cardiovascular events in diabetic patients [22]. These medications may offer protective effects against the heightened cardiovascular risks associated with COVID-19 in this high-risk population.

Furthermore, rapid structural changes to the healthcare system to adapt to the massive influx of SARS-CoV-2 infection have also been found to harm the level of care provided to patients with cardiovascular disease amongst others. As a result of these changes several elective procedures were deferred to reduce the risk of infection to patients and healthcare workers. Consequently, several patients delayed seeking primary intervention upon onset of symptoms. This has been reported as increased door-to-device times and decreased hospital admission rates by 40–50 %. This resulted in increased emergency admissions and mortality as the number of prehospital deaths and out-of-hospital cardiac arrests rose [17,[23], [24], [25]]. The impact of COVID-19 may also be extended to racial differences demonstrated in mortality. The NH Black population with coronary vascular disease has been linked to higher mortality rates when infected with COVID 19 which is consistent with our findings. This may be a result of different pathophysiology of the disease, unfavorable socioeconomic backgrounds, lack of access to healthcare, and reduced health-seeking behaviors [26].

The results also determined that men with diabetes had a higher mortality due to atherosclerotic disease than women. These results can be due to a multitude of risk factors such as the cardioprotective role of estrogen against the inflammatory processes and vascular responses that lead to the development of atherosclerosis. As per past literature, differences in plaque morphology also result in lower rates of diagnoses of atherosclerotic disease in women as they have diffusely spread plaques that minimally occlude blood flow, which is difficult to diagnose via angiography [27,28]. These morphologic and etiologic variations between sexes are overlooked in diagnostic criteria and management, which usually lead to lower reported rates of mortality in women. Underdiagnosis of atherosclerotic disease in women may also be a result of atypical presentations such as shortness of breath or gastrointestinal symptoms with no chest pain. A lack of awareness regarding sex-specific risk factors and inadequate screening practices have also contributed to the lower rates of atherosclerotic disease in women [27,29].

In addition, the results also indicates that Black diabetic patients have a higher rate of mortality due to atherosclerosis than any other racial group. These inequalities can be explained by a complex interplay of multitudinous elements. Black individuals with diabetes often present with multiple comorbid conditions that exacerbate their cardiovascular risk profiles. Notably, the prevalence of hypertension is significantly higher among Black diabetics, which is a critical risk factor for atherosclerosis [30]. The odds of hospitalization due to ASCVD markedly increase with these comorbidities, highlighting a compounded risk profile. Additionally, emerging research suggests that non-traditional cardiovascular risk factors, such as subclinical inflammation, may play a more significant role in the elevated rates of cardiovascular disease observed in Black populations [31]. This subclinical inflammation can accelerate the progression of atherosclerosis, leading to worse outcomes in diabetic patients [32]. Furthermore, Black individuals tend to exhibit greater insulin resistance and hyperinsulinemia compared to their white counterparts, a metabolic difference that contributes to more severe complications from diabetes, including increased cardiovascular morbidity and mortality [33].

The data further revealed higher mortality rates in non-metropolitan areas compared to metropolitan areas. Rural areas are home to around one-fifth of the American population and poor cardiovascular outcomes there could reflect poorer cardiovascular risk profile and access to healthcare [34]. Additionally, these results can be attributed to a complex interplay of multiple factors, such as economic and demographical changes over the years. Recent literature has revealed that non-metropolitan areas in the US have experienced economic decline and reductions in population [34]. Furthermore, since the COVID-19 pandemic, rural hospitals have been facing closures at an alarming rate, indicating further inequalities between rural and urban areas [34]. For racial and ethnic minorities, structural inequities are magnified further in rural areas. Moreover, atherosclerosis mortality rates due to diabetes were comparatively higher in the western census regions. This can be attributed to a multitude of factors, such as the availability of top-tier healthcare services, socioeconomic conditions, lifestyle choices, and genetic predisposition of the locals.

## 5 Future directions

Future research should expand in several additional directions to comprehensively address the increasing mortality trends linked to atherosclerosis in diabetic patients. One key area is the exploration of genetic and molecular biomarkers that may predispose individuals to accelerated atherosclerosis progression in the context of diabetes [35]. Understanding these genetic influences can guide the development of targeted therapies, including precision medicine approaches, tailored to individuals at higher risk. Another avenue involves increasing awareness regarding the impact of lifestyle interventions, such as dietary modifications, physical activity, and weight management, on slowing or reversing atherosclerotic changes in diabetic patients [36].

Moreover, the role of social determinants of health, including socioeconomic status, education, and access to healthcare, should be further investigated to elucidate their contribution to the observed mortality disparities. Addressing these determinants through community-based interventions and public health initiatives can help reduce health inequities. Emerging technologies, such as artificial intelligence (AI) and machine learning (ML), offer exciting prospects for predictive modeling, enabling early identification of high-risk individuals based on clinical and non-clinical factors [37,38].

Finally, a focus on the long-term impact of novel therapeutic approaches such as gene editing (e.g., CRISPR-based therapies) and RNA interference (RNAi) for modulating lipid metabolism and inflammatory pathways should be pursued [39]. These cutting-edge therapies hold promise for altering the disease trajectory in atherosclerosis and diabetes. Additionally, addressing the cost-effectiveness and real-world applicability of these interventions in both high-income and resource-limited settings will be essential to ensure equitable access and broad implementation.

## 6 Limitations

Several limitations should be considered. First, the physician may be unable to appropriately diagnose the disease during the patient's lifetime, misdiagnose the patient, or incorrectly enter the ICD codes which may lead to underestimation or misclassification of atherosclerosis and diabetes as a cause of death. Second, the data available may lack information about the social determinants of available healthcare, which may influence demographic variations pertaining to atherosclerosis-related mortality due to diabetes. Third, the data on the medical treatments and interventions is not available. Last, the database fails to provide data relevant to clinical variables including vital signs, laboratory findings, genetic analysis, and echocardiographic data that may prove helpful in understanding the phenotypic variations in atherosclerosis and diabetes patients.

## 7 Conclusion

The results revealed a decline in mortality rates from 1999 to 2018 followed by a rapid rise increase till 2020 in the United States of America. Nonetheless, disparities persist among gender, race, age, geographical and demographical regions, and the predisposing factors to cardiovascular diseases, particularly diabetes. Notably, adult men exhibited higher AAMRs as compared to adult women, NH Blacks were found to have the highest AAMRs as compared to other racial groups and non-metropolitan areas were consistent with having higher AAMRs as compared to other regions. Addressing these disparities requires targeted and uniform health policies aimed at reducing atherosclerosis-related mortality in diabetic adults, emphasizing early prevention, diagnosis, and compliance to treatment and prompt referrals. It is imperative to note that the governmental health reforms should be implemented equally in all regions and proper guidelines and measures must be taken as was consistent during the COVID-19 pandemic. Moreover, educating the masses at the community level will ensure a decline in such diseases.

## Author contributions

• Conception or design of the work: FL.

• Acquisition, analysis, or interpretation of data for the work: YHA.

• Drafting of the manuscript: YHA, FL, HA, FH.

• Reviewing and editing of the manuscript: FL, RA, AH, VJ.

## Data availability

The data underlying this article are available in CDC WONDER Database, at https://wonder.cdc.gov/mcd-icd10.html.

## Funding

None to declare.

## Declaration of competing interest

The authors declare that they have no known competing financial interests or personal relationships that could have appeared to influence the work reported in this paper.
