# Supplementary material for: Trends of atherosclerosis-related mortality in adults with diabetes: A cross-sectional analysis of U.S. national data
Source: Atheroscler Plus. 2025 May 2;60:27–34. doi: 10.1016/j.athplu.2025.04.001 (PMC12140960; doi:10.1016/j.athplu.2025.04.001)
Supplement: Multimedia component 1 [file mmc1.docx]

**Supplemental Materials:** Trends of Atherosclerosis-Related Mortality in Adults with Diabetes: A Cross-Sectional Analysis of U.S. National Data

**Figures:**

**Supplementary Figure 1** Atherosclerosis-related mortality trends in adults with diabetes population stratified across 50 States in 1999-2020.

**Supplementary Figure 2** Atherosclerosis-related mortality trends in adults with diabetes stratified for census regions in 1999-2020

**Tables:**

**Supplemental Table 1** Atherosclerosis-related Deaths, Stratified by Sex and Race, in Diabetic Patients in the United States, 1999 to 2020

**Supplemental Table 2** Atherosclerosis-related Mortality, Stratified by Place of Death in Diabetic Patients in the United States, 1999 to 2020

**Supplemental Table 3** Atherosclerosis-related Age-Adjusted Mortality Rates per 100,000, Stratified in Diabetic Patients in the United States, 1999 to 2020

**Supplemental Table 4** Atherosclerosis-related Age-Adjusted Mortality Rates per 100,000, Stratified by Sex in Diabetic Patients in the United States, 1999 to 2020

**Supplemental Table 5** Atherosclerosis-related Age-Adjusted Mortality Rates per 100,000, Stratified by Race in Diabetic Patients in the United States, 1999 to 2020

**Supplemental Table 6** Atherosclerosis-related Age-Adjusted Mortality Rates per 100,000, Stratified by Urban-Rural Classification in Diabetic Patients in the United States, 1999 to 2020

**Supplemental Table 7** Atherosclerosis-related Age-Adjusted Mortality Rates per 100,000, Stratified by States in Diabetic Patients in the United States, 1999 to 2020

**Supplemental Table 8** Atherosclerosis-related Age-Adjusted Mortality Rates per 100,000, Stratified by Census Regions in Diabetic Patients in the United States, 1999 to 2020

**Supplementary Figure 1** Atherosclerosis-related mortality trends in adults with diabetes population stratified across 50 States in 1999-2020.

**
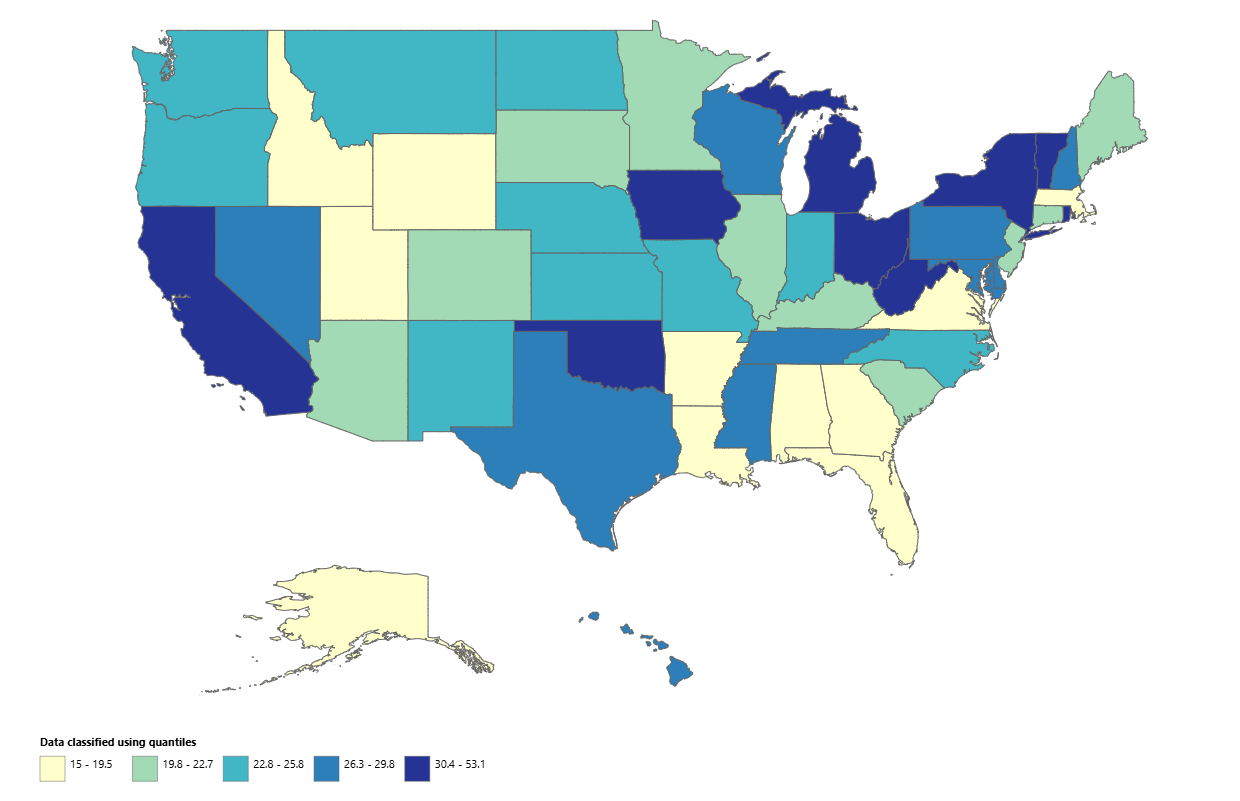
**

**Supplementary Figure 2** Atherosclerosis-related mortality trends in adults with diabetes stratified for census regions in 1999-2020

**
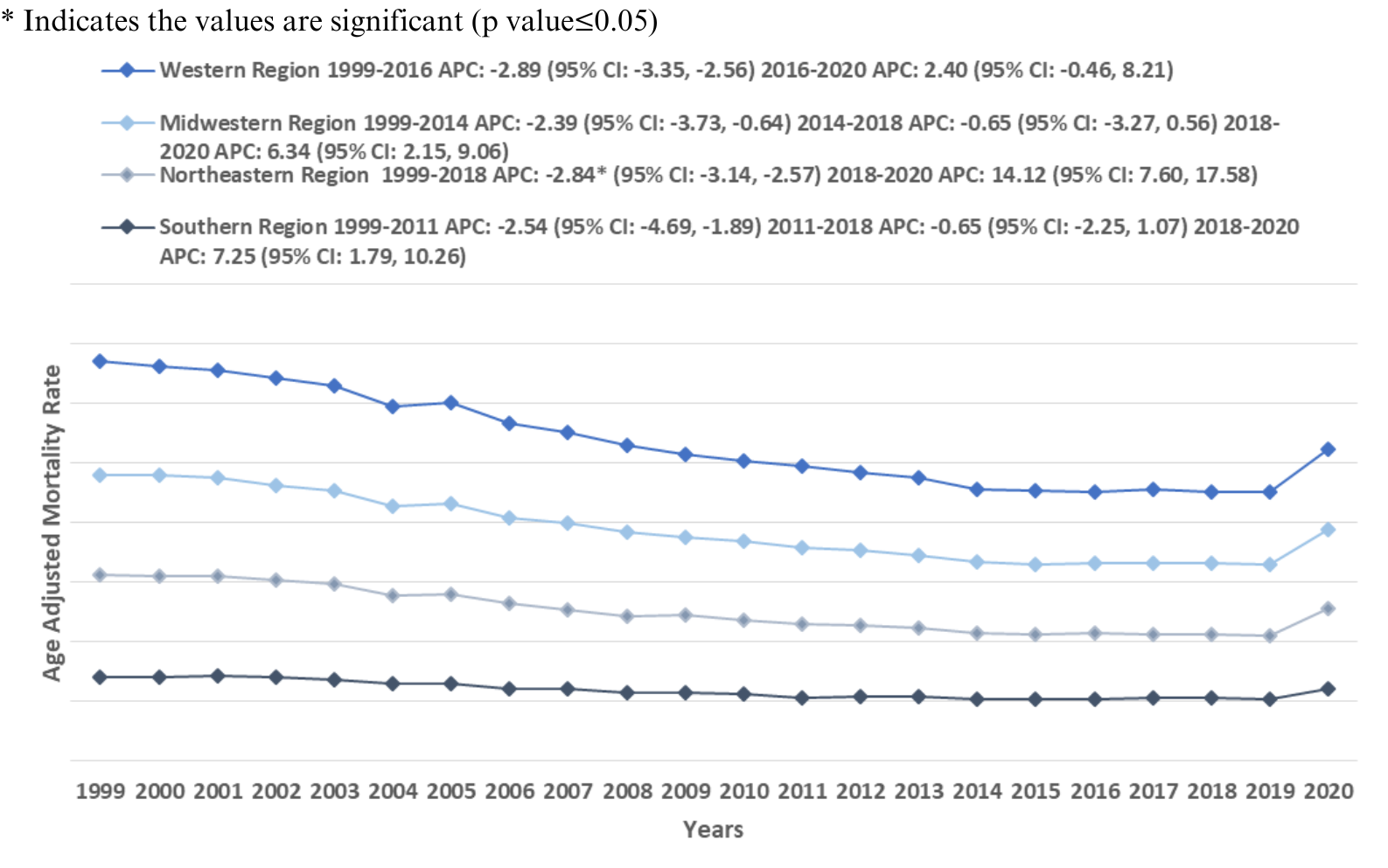
**

**Supplemental Table 1** Atherosclerosis-related Deaths, Stratified by Sex and Race, in Diabetic Patients in the United States, 1999 to 2020

|  | Deaths (n)  n = X | | | | | | | |
| --- | --- | --- | --- | --- | --- | --- | --- | --- |
| Year | **Overall** | **Women** | **Men** | **NH White** | **NH Black or African American** | **NH Asian or Pacific Islander** | **NH American Indian or Alaska Native** | **Hispanic or Latino** |
| 1999 | 31170 | 15649 | 15521 | 24572 | 3709 | 644 | 159 | 1979 |
| 2000 | 31257 | 15433 | 15824 | 24632 | 3846 | 600 | 157 | 1914 |
| 2001 | 31574 | 15314 | 16260 | 24579 | 4006 | 633 | 167 | 2082 |
| 2002 | 31487 | 15065 | 16422 | 24331 | 4062 | 705 | 179 | 2102 |
| 2003 | 31300 | 14699 | 16601 | 24002 | 4127 | 718 | 195 | 2146 |
| 2004 | 30106 | 14039 | 16067 | 23041 | 3927 | 696 | 173 | 2174 |
| 2005 | 30985 | 14263 | 16722 | 23521 | 4109 | 744 | 187 | 2354 |
| 2006 | 29857 | 13369 | 16488 | 22554 | 3974 | 738 | 188 | 2327 |
| 2007 | 29749 | 13072 | 16677 | 22506 | 3963 | 722 | 212 | 2296 |
| 2008 | 29168 | 12819 | 16349 | 22086 | 3826 | 781 | 178 | 2241 |
| 2009 | 28989 | 12378 | 16611 | 21844 | 3749 | 758 | 232 | 2323 |
| 2010 | 28760 | 12371 | 16389 | 21497 | 3681 | 810 | 227 | 2438 |
| 2011 | 29086 | 12113 | 16973 | 21700 | 3819 | 802 | 223 | 2461 |
| 2012 | 29302 | 11899 | 17403 | 21857 | 3796 | 884 | 237 | 2421 |
| 2013 | 29653 | 11817 | 17836 | 21867 | 3897 | 894 | 269 | 2596 |
| 2014 | 29225 | 11429 | 17796 | 21386 | 3847 | 964 | 282 | 2622 |
| 2015 | 29611 | 11361 | 18250 | 21555 | 3943 | 986 | 284 | 2662 |
| 2016 | 30148 | 11413 | 18735 | 21805 | 4081 | 1025 | 292 | 2827 |
| 2017 | 31280 | 11683 | 19597 | 22484 | 4267 | 1101 | 287 | 3000 |
| 2018 | 31664 | 11693 | 19971 | 22805 | 4223 | 1157 | 324 | 3016 |
| 2019 | 32248 | 11918 | 20330 | 23245 | 4308 | 1173 | 293 | 3123 |
| 2020 | 37963 | 13745 | 24218 | 26239 | 5685 | 1517 | 331 | 4055 |
| Total | 674582 | 287542 | 387040 | 504108 | 88845 | 19052 | 5076 | 55159 |

NH, non-Hispanic.

**Supplemental Table 2** Atherosclerosis-related Mortality, Stratified by Place of Death in Diabetic Patients in the United States, 1999 to 2020

|  | Deaths | | | |  |  |
| --- | --- | --- | --- | --- | --- | --- |
| Year | **Medical Facility** | **Nursing Home/Long-term Care Facility** | **Hospices** | **Home** | **Unknown** | **Others** |
| 1999 | 15181 | 7283 | 31 | 7879 | 13 | 820 |
| 2000 | 14843 | 7487 | 62 | 8017 | 78 | 904 |
| 2001 | 14802 | 7438 | 173 | 8321 | 66 | 1000 |
| 2002 | 14325 | 7525 | 247 | 8535 | 78 | 1095 |
| 2003 | 13844 | 7253 | 392 | 8989 | 79 | 1105 |
| 2004 | 12984 | 7065 | 442 | 8766 | 50 | 1163 |
| 2005 | 12883 | 7176 | 475 | 9428 | 371 | 1247 |
| 2006 | 12367 | 6644 | 536 | 9361 | 562 | 1159 |
| 2007 | 12000 | 6608 | 633 | 9502 | 22 | 1197 |
| 2008 | 11184 | 6302 | 696 | 9698 | 15 | 1171 |
| 2009 | 10738 | 6152 | 786 | 9933 | 20 | 1129 |
| 2010 | 10760 | 5967 | 758 | 10161 | 11 | 1314 |
| 2011 | 10538 | 5933 | 926 | 10639 | 34 | 1328 |
| 2012 | 10369 | 5774 | 971 | 11066 | 14 | 1377 |
| 2013 | 10193 | 5565 | 970 | 11615 | 10 | 1483 |
| 2014 | 9865 | 5251 | 1010 | 11905 | 1471 | 1412 |
| 2015 | 9726 | 5290 | 1133 | 12218 | 13 | 1437 |
| 2016 | 9681 | 5161 | 1231 | 12800 | 78 | 1527 |
| 2017 | 9987 | 5314 | 11472 | 13393 | 66 | 1606 |
| 2018 | 9897 | 5252 | 31 | 13903 | 78 | 1596 |
| 2019 | 9717 | 5344 | 62 | 14248 | 79 | 1800 |
| 2020 | 10309 | 5577 | 173 | 18690 | 50 | 2148 |
| Total | 256193 | 137361 | 247 | 239067 | 371 | 29018 |

**Supplemental Table 3** Atherosclerosis-related Age-Adjusted Mortality Rates per 100,000, Stratified in Diabetic Patients in the United States, 1999 to 2020

| Year | Age Adjusted Rate | Age Adjusted Rate  Lower 95% CI | Age Adjusted Rate  Upper 95% CI |
| --- | --- | --- | --- |
| 1999 | 32.8 | 32.4 | 33.1 |
| 2000 | 32.4 | 32.1 | 32.8 |
| 2001 | 32.2 | 31.9 | 32.6 |
| 2002 | 31.6 | 31.3 | 32 |
| 2003 | 30.9 | 30.5 | 31.2 |
| 2004 | 29.2 | 28.9 | 29.6 |
| 2005 | 29.5 | 29.2 | 29.8 |
| 2006 | 27.8 | 27.5 | 28.1 |
| 2007 | 27.2 | 26.9 | 27.5 |
| 2008 | 26 | 25.7 | 26.3 |
| 2009 | 25.4 | 25.1 | 25.7 |
| 2010 | 24.7 | 24.5 | 25 |
| 2011 | 24.3 | 24 | 24.6 |
| 2012 | 23.8 | 23.5 | 24.1 |
| 2013 | 23.5 | 23.3 | 23.8 |
| 2014 | 22.6 | 22.3 | 22.9 |
| 2015 | 22.4 | 22.2 | 22.7 |
| 2016 | 22.3 | 22.1 | 22.6 |
| 2017 | 22.6 | 22.4 | 22.9 |
| 2018 | 22.4 | 22.2 | 22.7 |
| 2019 | 22.3 | 22.1 | 22.6 |
| 2020 | 25.8 | 25.6 | 26.1 |

**Supplemental Table 4** Atherosclerosis-related Age-Adjusted Mortality Rates per 100,000, Stratified by Sex in Diabetic Patients in the United States, 1999 to 2020

| Gender | Year | Age Adjusted Rate | Age Adjusted Rate  Lower 95% CI | Age Adjusted Rate  Upper 95% CI |
| --- | --- | --- | --- | --- |
| Female | 1999 | 27.1 | 26.7 | 27.6 |
| Female | 2000 | 26.4 | 26 | 26.8 |
| Female | 2001 | 25.9 | 25.4 | 26.3 |
| Female | 2002 | 25.1 | 24.7 | 25.5 |
| Female | 2003 | 24.2 | 23.8 | 24.6 |
| Female | 2004 | 22.9 | 22.5 | 23.3 |
| Female | 2005 | 22.9 | 22.5 | 23.2 |
| Female | 2006 | 21.1 | 20.7 | 21.4 |
| Female | 2007 | 20.2 | 19.8 | 20.5 |
| Female | 2008 | 19.4 | 19.1 | 19.8 |
| Female | 2009 | 18.5 | 18.2 | 18.8 |
| Female | 2010 | 18.1 | 17.8 | 18.4 |
| Female | 2011 | 17.3 | 17 | 17.7 |
| Female | 2012 | 16.7 | 16.4 | 17 |
| Female | 2013 | 16.3 | 16 | 16.6 |
| Female | 2014 | 15.4 | 15.1 | 15.7 |
| Female | 2015 | 15.1 | 14.8 | 15.3 |
| Female | 2016 | 14.8 | 14.6 | 15.1 |
| Female | 2017 | 14.8 | 14.5 | 15.1 |
| Female | 2018 | 14.6 | 14.4 | 14.9 |
| Female | 2019 | 14.6 | 14.3 | 14.8 |
| Female | 2020 | 16.5 | 16.3 | 16.8 |
| Male | 1999 | 40.5 | 39.8 | 41.1 |
| Male | 2000 | 40.9 | 40.2 | 41.5 |
| Male | 2001 | 40.9 | 40.3 | 41.5 |
| Male | 2002 | 40.6 | 40 | 41.3 |
| Male | 2003 | 40.1 | 39.5 | 40.7 |
| Male | 2004 | 38.1 | 37.5 | 38.7 |
| Male | 2005 | 38.5 | 38 | 39.1 |
| Male | 2006 | 37 | 36.4 | 37.6 |
| Male | 2007 | 36.5 | 35.9 | 37.1 |
| Male | 2008 | 34.9 | 34.3 | 35.4 |
| Male | 2009 | 34.5 | 34 | 35.1 |
| Male | 2010 | 33.5 | 33 | 34 |
| Male | 2011 | 33.5 | 33 | 34 |
| Male | 2012 | 33.2 | 32.7 | 33.7 |
| Male | 2013 | 33 | 32.5 | 33.5 |
| Male | 2014 | 31.9 | 31.4 | 32.4 |
| Male | 2015 | 31.9 | 31.4 | 32.4 |
| Male | 2016 | 32 | 31.5 | 32.4 |
| Male | 2017 | 32.5 | 32 | 33 |
| Male | 2018 | 32.3 | 31.9 | 32.8 |
| Male | 2019 | 32.2 | 31.7 | 32.6 |
| Male | 2020 | 37.5 | 37 | 38 |

**Supplemental Table 5** Atherosclerosis-related Age-Adjusted Mortality Rates per 100,000, Stratified by Race in Diabetic Patients in the United States, 1999 to 2020

| Race | Year | Age Adjusted Rate | Age Adjusted Rate  Lower 95% CI | Age Adjusted Rate  Upper 95% CI |
| --- | --- | --- | --- | --- |
| NH American Indian or Alaska Native | 1999 | 40.5 | 33.8 | 47.2 |
| NH American Indian or Alaska Native | 2000 | 35.4 | 29.6 | 41.3 |
| NH American Indian or Alaska Native | 2001 | 39.2 | 33 | 45.4 |
| NH American Indian or Alaska Native | 2002 | 40.8 | 34.5 | 47.1 |
| NH American Indian or Alaska Native | 2003 | 42.3 | 36 | 48.7 |
| NH American Indian or Alaska Native | 2004 | 36.3 | 30.5 | 42.1 |
| NH American Indian or Alaska Native | 2005 | 37.3 | 31.5 | 43 |
| NH American Indian or Alaska Native | 2006 | 34.9 | 29.6 | 40.2 |
| NH American Indian or Alaska Native | 2007 | 40.1 | 34.4 | 45.9 |
| NH American Indian or Alaska Native | 2008 | 31.4 | 26.5 | 36.4 |
| NH American Indian or Alaska Native | 2009 | 38.7 | 33.3 | 44.1 |
| NH American Indian or Alaska Native | 2010 | 38.4 | 33.1 | 43.8 |
| NH American Indian or Alaska Native | 2011 | 33.9 | 29.1 | 38.6 |
| NH American Indian or Alaska Native | 2012 | 35.6 | 30.8 | 40.4 |
| NH American Indian or Alaska Native | 2013 | 36 | 31.4 | 40.6 |
| NH American Indian or Alaska Native | 2014 | 36.6 | 32.1 | 41 |
| NH American Indian or Alaska Native | 2015 | 36.7 | 32.2 | 41.2 |
| NH American Indian or Alaska Native | 2016 | 36.4 | 32 | 40.8 |
| NH American Indian or Alaska Native | 2017 | 34.1 | 30 | 38.2 |
| NH American Indian or Alaska Native | 2018 | 36.5 | 32.4 | 40.6 |
| NH American Indian or Alaska Native | 2019 | 31 | 27.3 | 34.6 |
| NH American Indian or Alaska Native | 2020 | 34.5 | 30.6 | 38.3 |
| NH Asian or Pacific Islander | 1999 | 31 | 28.5 | 33.5 |
| NH Asian or Pacific Islander | 2000 | 26.7 | 24.5 | 28.9 |
| NH Asian or Pacific Islander | 2001 | 26.3 | 24.2 | 28.4 |
| NH Asian or Pacific Islander | 2002 | 27.4 | 25.3 | 29.5 |
| NH Asian or Pacific Islander | 2003 | 26.1 | 24.1 | 28.1 |
| NH Asian or Pacific Islander | 2004 | 24.1 | 22.2 | 25.9 |
| NH Asian or Pacific Islander | 2005 | 24 | 22.2 | 25.8 |
| NH Asian or Pacific Islander | 2006 | 22.3 | 20.6 | 23.9 |
| NH Asian or Pacific Islander | 2007 | 20.7 | 19.2 | 22.3 |
| NH Asian or Pacific Islander | 2008 | 21.4 | 19.8 | 22.9 |
| NH Asian or Pacific Islander | 2009 | 19.7 | 18.3 | 21.1 |
| NH Asian or Pacific Islander | 2010 | 20.4 | 18.9 | 21.8 |
| NH Asian or Pacific Islander | 2011 | 18.3 | 17 | 19.6 |
| NH Asian or Pacific Islander | 2012 | 19 | 17.7 | 20.3 |
| NH Asian or Pacific Islander | 2013 | 17.8 | 16.6 | 18.9 |
| NH Asian or Pacific Islander | 2014 | 18 | 16.9 | 19.2 |
| NH Asian or Pacific Islander | 2015 | 17.5 | 16.4 | 18.6 |
| NH Asian or Pacific Islander | 2016 | 16.9 | 15.8 | 17.9 |
| NH Asian or Pacific Islander | 2017 | 17.1 | 16.1 | 18.1 |
| NH Asian or Pacific Islander | 2018 | 17 | 16 | 18 |
| NH Asian or Pacific Islander | 2019 | 16.7 | 15.7 | 17.7 |
| NH Asian or Pacific Islander | 2020 | 20.3 | 19.3 | 21.4 |
| NH Black or African American | 1999 | 46.3 | 44.8 | 47.8 |
| NH Black or African American | 2000 | 47.5 | 46 | 49.1 |
| NH Black or African American | 2001 | 48.5 | 47 | 50 |
| NH Black or African American | 2002 | 48.1 | 46.6 | 49.6 |
| NH Black or African American | 2003 | 48.1 | 46.6 | 49.6 |
| NH Black or African American | 2004 | 44.8 | 43.4 | 46.2 |
| NH Black or African American | 2005 | 45 | 43.6 | 46.4 |
| NH Black or African American | 2006 | 42.5 | 41.2 | 43.9 |
| NH Black or African American | 2007 | 41.3 | 40 | 42.6 |
| NH Black or African American | 2008 | 38.5 | 37.3 | 39.8 |
| NH Black or African American | 2009 | 36.9 | 35.7 | 38.2 |
| NH Black or African American | 2010 | 35.2 | 34 | 36.4 |
| NH Black or African American | 2011 | 35.1 | 33.9 | 36.2 |
| NH Black or African American | 2012 | 33.6 | 32.5 | 34.7 |
| NH Black or African American | 2013 | 33.3 | 32.2 | 34.4 |
| NH Black or African American | 2014 | 31.4 | 30.4 | 32.5 |
| NH Black or African American | 2015 | 31.1 | 30.1 | 32.1 |
| NH Black or African American | 2016 | 31.4 | 30.4 | 32.4 |
| NH Black or African American | 2017 | 31.7 | 30.7 | 32.7 |
| NH Black or African American | 2018 | 30.3 | 29.4 | 31.2 |
| NH Black or African American | 2019 | 30.4 | 29.5 | 31.3 |
| NH Black or African American | 2020 | 39 | 38 | 40.1 |
| NH White | 1999 | 30.6 | 30.2 | 31 |
| NH White | 2000 | 30.4 | 30 | 30.8 |
| NH White | 2001 | 30 | 29.6 | 30.4 |
| NH White | 2002 | 29.3 | 28.9 | 29.7 |
| NH White | 2003 | 28.6 | 28.2 | 28.9 |
| NH White | 2004 | 27.1 | 26.7 | 27.4 |
| NH White | 2005 | 27.2 | 26.9 | 27.6 |
| NH White | 2006 | 25.8 | 25.4 | 26.1 |
| NH White | 2007 | 25.3 | 25 | 25.6 |
| NH White | 2008 | 24.4 | 24 | 24.7 |
| NH White | 2009 | 23.8 | 23.4 | 24.1 |
| NH White | 2010 | 23.1 | 22.8 | 23.4 |
| NH White | 2011 | 22.8 | 22.5 | 23.1 |
| NH White | 2012 | 22.5 | 22.2 | 22.8 |
| NH White | 2013 | 22.1 | 21.8 | 22.4 |
| NH White | 2014 | 21.3 | 21 | 21.6 |
| NH White | 2015 | 21.2 | 20.9 | 21.5 |
| NH White | 2016 | 21 | 20.7 | 21.3 |
| NH White | 2017 | 21.3 | 21 | 21.6 |
| NH White | 2018 | 21.3 | 21 | 21.6 |
| NH White | 2019 | 21.3 | 21.1 | 21.6 |
| NH White | 2020 | 23.8 | 23.5 | 24.1 |
| Hispanic or Latino | 1999 | 43 | 41.1 | 45 |
| Hispanic or Latino | 2000 | 40.4 | 38.5 | 42.3 |
| Hispanic or Latino | 2001 | 40.9 | 39.1 | 42.7 |
| Hispanic or Latino | 2002 | 39 | 37.3 | 40.7 |
| Hispanic or Latino | 2003 | 37.9 | 36.3 | 39.6 |
| Hispanic or Latino | 2004 | 36.4 | 34.8 | 38 |
| Hispanic or Latino | 2005 | 37.2 | 35.7 | 38.8 |
| Hispanic or Latino | 2006 | 34.6 | 33.2 | 36.1 |
| Hispanic or Latino | 2007 | 32.5 | 31.1 | 33.9 |
| Hispanic or Latino | 2008 | 30.4 | 29.1 | 31.7 |
| Hispanic or Latino | 2009 | 29.5 | 28.2 | 30.7 |
| Hispanic or Latino | 2010 | 30.2 | 29 | 31.5 |
| Hispanic or Latino | 2011 | 28 | 26.8 | 29.1 |
| Hispanic or Latino | 2012 | 26.1 | 25 | 27.2 |
| Hispanic or Latino | 2013 | 26.7 | 25.6 | 27.7 |
| Hispanic or Latino | 2014 | 25.1 | 24.1 | 26.1 |
| Hispanic or Latino | 2015 | 24 | 23 | 24.9 |
| Hispanic or Latino | 2016 | 24.4 | 23.5 | 25.4 |
| Hispanic or Latino | 2017 | 24.6 | 23.7 | 25.5 |
| Hispanic or Latino | 2018 | 23.6 | 22.7 | 24.5 |
| Hispanic or Latino | 2019 | 23.2 | 22.3 | 24 |
| Hispanic or Latino | 2020 | 29 | 28 | 29.9 |

NH = non-Hispanic.

**Supplemental Table 6** Atherosclerosis-related Age-Adjusted Mortality Rates per 100,000, Stratified by Urban-Rural Classification in Diabetic Patients in the United States, 1999 to 2020

| Metropolitan vs. non-metropolitan area | Year | Age Adjusted Rate | Age Adjusted Rate  Lower 95% CI | Age Adjusted Rate  Upper 95% CI |
| --- | --- | --- | --- | --- |
| Metropolitan | 1999 | 33.1 | 32.7 | 33.5 |
| Metropolitan | 2000 | 32.8 | 32.4 | 33.2 |
| Metropolitan | 2001 | 32.5 | 32.1 | 32.9 |
| Metropolitan | 2002 | 31.8 | 31.4 | 32.2 |
| Metropolitan | 2003 | 31 | 30.7 | 31.4 |
| Metropolitan | 2004 | 29.4 | 29 | 29.8 |
| Metropolitan | 2005 | 29.6 | 29.3 | 30 |
| Metropolitan | 2006 | 28 | 27.6 | 28.3 |
| Metropolitan | 2007 | 27.2 | 26.8 | 27.5 |
| Metropolitan | 2008 | 25.9 | 25.6 | 26.3 |
| Metropolitan | 2009 | 25.3 | 24.9 | 25.6 |
| Metropolitan | 2010 | 24.7 | 24.4 | 25 |
| Metropolitan | 2011 | 24.2 | 23.9 | 24.5 |
| Metropolitan | 2012 | 23.6 | 23.3 | 23.9 |
| Metropolitan | 2013 | 23.4 | 23.1 | 23.7 |
| Metropolitan | 2014 | 22.3 | 22 | 22.6 |
| Metropolitan | 2015 | 22 | 21.7 | 22.3 |
| Metropolitan | 2016 | 22 | 21.7 | 22.3 |
| Metropolitan | 2017 | 22.4 | 22.1 | 22.6 |
| Metropolitan | 2018 | 22 | 21.7 | 22.3 |
| Metropolitan | 2019 | 21.7 | 21.5 | 22 |
| Metropolitan | 2020 | 25.5 | 25.2 | 25.7 |
| Non-metropolitan | 1999 | 31 | 30.2 | 31.8 |
| Non-metropolitan | 2000 | 31 | 30.2 | 31.9 |
| Non-metropolitan | 2001 | 31.1 | 30.3 | 31.9 |
| Non-metropolitan | 2002 | 30.7 | 29.9 | 31.5 |
| Non-metropolitan | 2003 | 30.2 | 29.4 | 31 |
| Non-metropolitan | 2004 | 28.5 | 27.7 | 29.3 |
| Non-metropolitan | 2005 | 28.7 | 27.9 | 29.5 |
| Non-metropolitan | 2006 | 27.1 | 26.4 | 27.8 |
| Non-metropolitan | 2007 | 27 | 26.3 | 27.7 |
| Non-metropolitan | 2008 | 26.7 | 25.9 | 27.4 |
| Non-metropolitan | 2009 | 25.7 | 25 | 26.4 |
| Non-metropolitan | 2010 | 25 | 24.3 | 25.7 |
| Non-metropolitan | 2011 | 24.8 | 24.1 | 25.5 |
| Non-metropolitan | 2012 | 24.9 | 24.3 | 25.6 |
| Non-metropolitan | 2013 | 24.4 | 23.7 | 25.1 |
| Non-metropolitan | 2014 | 24 | 23.3 | 24.6 |
| Non-metropolitan | 2015 | 24.6 | 23.9 | 25.3 |
| Non-metropolitan | 2016 | 23.8 | 23.2 | 24.5 |
| Non-metropolitan | 2017 | 24 | 23.4 | 24.7 |
| Non-metropolitan | 2018 | 24.4 | 23.7 | 25 |
| Non-metropolitan | 2019 | 25.3 | 24.6 | 25.9 |
| Non-metropolitan | 2020 | 27.7 | 27 | 28.4 |

**Supplemental Table 7** Atherosclerosis-related Age-Adjusted Mortality Rates per 100,000, Stratified by States in Diabetic Patients in the United States, 1999 to 2020

| State | Age Adjusted Rate | Age Adjusted Rate  Lower 95% CI | Age Adjusted Rate  Upper 95% CI |
| --- | --- | --- | --- |
| Alabama | 16.5 | 16.2 | 16.9 |
| Alaska | 18.4 | 17 | 19.9 |
| Arizona | 19.8 | 19.4 | 20.1 |
| Arkansas | 16.5 | 16 | 17 |
| California | 34.8 | 34.6 | 35 |
| Colorado | 20.3 | 19.8 | 20.8 |
| Connecticut | 22 | 21.5 | 22.5 |
| Delaware | 27.9 | 26.7 | 29.1 |
| District of Columbia | 53.1 | 51 | 55.3 |
| Florida | 17.3 | 17.2 | 17.5 |
| Georgia | 17.9 | 17.5 | 18.2 |
| Hawaii | 26.7 | 25.7 | 27.6 |
| Idaho | 17.7 | 16.9 | 18.5 |
| Illinois | 21.1 | 20.8 | 21.3 |
| Indiana | 23 | 22.6 | 23.4 |
| Iowa | 33.9 | 33.2 | 34.6 |
| Kansas | 22.9 | 22.3 | 23.5 |
| Kentucky | 22.7 | 22.2 | 23.2 |
| Louisiana | 19.5 | 19.1 | 20 |
| Maine | 20.4 | 19.6 | 21.2 |
| Maryland | 27.7 | 27.2 | 28.2 |
| Massachusetts | 17.1 | 16.8 | 17.4 |
| Michigan | 33.4 | 33 | 33.8 |
| Minnesota | 22.3 | 21.8 | 22.7 |
| Mississippi | 28.7 | 28 | 29.4 |
| Missouri | 22.8 | 22.4 | 23.2 |
| Montana | 23 | 22 | 24 |
| Nebraska | 25.8 | 25 | 26.5 |
| Nevada | 29.3 | 28.5 | 30.1 |
| New Hampshire | 26.3 | 25.4 | 27.2 |
| New Jersey | 20.2 | 19.8 | 20.5 |
| New Mexico | 24.5 | 23.8 | 25.3 |
| New York | 32.4 | 32.1 | 32.6 |
| North Carolina | 25 | 24.7 | 25.4 |
| North Dakota | 24.8 | 23.5 | 26 |
| Ohio | 35.1 | 34.7 | 35.4 |
| Oklahoma | 44.7 | 44 | 45.5 |
| Oregon | 25.3 | 24.8 | 25.9 |
| Pennsylvania | 26.8 | 26.5 | 27.1 |
| Rhode Island | 30.4 | 29.3 | 31.4 |
| South Carolina | 21.4 | 21 | 21.9 |
| South Dakota | 20.9 | 19.9 | 22 |
| Tennessee | 29.8 | 29.3 | 30.3 |
| Texas | 28 | 27.8 | 28.3 |
| Utah | 15 | 14.4 | 15.6 |
| Vermont | 47.6 | 45.9 | 49.4 |
| Virginia | 18.2 | 17.9 | 18.6 |
| Washington | 25.2 | 24.8 | 25.6 |
| West Virginia | 37.3 | 36.4 | 38.2 |
| Wisconsin | 26.9 | 26.4 | 27.3 |
| Wyoming | 19.5 | 18.2 | 20.8 |

**Supplemental Table 8** Atherosclerosis-related Age-Adjusted Mortality Rates per 100,000, Stratified by Census Region in Diabetic Patients in the United States, 1999 to 2020

| Census Region | Year | Age Adjusted Rate | Age Adjusted Rate Lower 95% CI | Age Adjusted Rate Upper 95% CI |
| --- | --- | --- | --- | --- |
| Northeast | 1999 | 34.2 | 33.3 | 35 |
| Northeast | 2000 | 34.1 | 33.3 | 34.9 |
| Northeast | 2001 | 33.4 | 32.7 | 34.2 |
| Northeast | 2002 | 32.5 | 31.8 | 33.3 |
| Northeast | 2003 | 32.2 | 31.4 | 33 |
| Northeast | 2004 | 29.6 | 28.9 | 30.3 |
| Northeast | 2005 | 30.1 | 29.4 | 30.8 |
| Northeast | 2006 | 28.4 | 27.7 | 29.2 |
| Northeast | 2007 | 26.9 | 26.2 | 27.6 |
| Northeast | 2008 | 25.8 | 25.1 | 26.5 |
| Northeast | 2009 | 26 | 25.4 | 26.7 |
| Northeast | 2010 | 25.1 | 24.4 | 25.7 |
| Northeast | 2011 | 24.6 | 23.9 | 25.2 |
| Northeast | 2012 | 24 | 23.3 | 24.6 |
| Northeast | 2013 | 23 | 22.4 | 23.6 |
| Northeast | 2014 | 22 | 21.4 | 22.6 |
| Northeast | 2015 | 21.6 | 21 | 22.2 |
| Northeast | 2016 | 22.1 | 21.5 | 22.6 |
| Northeast | 2017 | 21.1 | 20.5 | 21.6 |
| Northeast | 2018 | 21.6 | 21.1 | 22.2 |
| Northeast | 2019 | 21.3 | 20.7 | 21.9 |
| Northeast | 2020 | 26.9 | 26.2 | 27.5 |
| Midwest | 1999 | 33.6 | 32.9 | 34.4 |
| Midwest | 2000 | 33.7 | 32.9 | 34.4 |
| Midwest | 2001 | 33 | 32.3 | 33.8 |
| Midwest | 2002 | 31.9 | 31.2 | 32.6 |
| Midwest | 2003 | 31.4 | 30.7 | 32.2 |
| Midwest | 2004 | 29.9 | 29.2 | 30.6 |
| Midwest | 2005 | 30.1 | 29.4 | 30.8 |
| Midwest | 2006 | 28.8 | 28.1 | 29.4 |
| Midwest | 2007 | 29.1 | 28.4 | 29.7 |
| Midwest | 2008 | 28.2 | 27.6 | 28.9 |
| Midwest | 2009 | 26.5 | 25.9 | 27.1 |
| Midwest | 2010 | 26.2 | 25.6 | 26.8 |
| Midwest | 2011 | 25.5 | 24.8 | 26.1 |
| Midwest | 2012 | 25.3 | 24.7 | 25.9 |
| Midwest | 2013 | 24.3 | 23.7 | 24.9 |
| Midwest | 2014 | 23.8 | 23.3 | 24.4 |
| Midwest | 2015 | 23.6 | 23 | 24.1 |
| Midwest | 2016 | 23.3 | 22.8 | 23.9 |
| Midwest | 2017 | 23.8 | 23.2 | 24.3 |
| Midwest | 2018 | 23.6 | 23 | 24.1 |
| Midwest | 2019 | 23.9 | 23.3 | 24.4 |
| Midwest | 2020 | 26.7 | 26.1 | 27.3 |
| South | 1999 | 28.1 | 27.5 | 28.6 |
| South | 2000 | 28.1 | 27.6 | 28.7 |
| South | 2001 | 28.6 | 28 | 29.2 |
| South | 2002 | 28.1 | 27.6 | 28.7 |
| South | 2003 | 27.3 | 26.8 | 27.9 |
| South | 2004 | 26.1 | 25.6 | 26.6 |
| South | 2005 | 26 | 25.5 | 26.5 |
| South | 2006 | 24.3 | 23.8 | 24.8 |
| South | 2007 | 24 | 23.6 | 24.5 |
| South | 2008 | 22.9 | 22.4 | 23.3 |
| South | 2009 | 22.8 | 22.3 | 23.3 |
| South | 2010 | 22.3 | 21.8 | 22.7 |
| South | 2011 | 21.3 | 20.8 | 21.7 |
| South | 2012 | 21.5 | 21 | 21.9 |
| South | 2013 | 21.7 | 21.3 | 22.1 |
| South | 2014 | 20.9 | 20.5 | 21.3 |
| South | 2015 | 20.9 | 20.5 | 21.3 |
| South | 2016 | 20.9 | 20.5 | 21.3 |
| South | 2017 | 21.3 | 20.9 | 21.7 |
| South | 2018 | 21 | 20.6 | 21.4 |
| South | 2019 | 20.9 | 20.5 | 21.3 |
| South | 2020 | 24.3 | 23.9 | 24.7 |
| West | 1999 | 38.5 | 37.6 | 39.4 |
| West | 2000 | 36.8 | 35.9 | 37.6 |
| West | 2001 | 36.3 | 35.5 | 37.2 |
| West | 2002 | 36.3 | 35.5 | 37.1 |
| West | 2003 | 35 | 34.1 | 35.8 |
| West | 2004 | 33.4 | 32.6 | 34.2 |
| West | 2005 | 34.1 | 33.4 | 34.9 |
| West | 2006 | 32.1 | 31.4 | 32.9 |
| West | 2007 | 30.5 | 29.7 | 31.2 |
| West | 2008 | 29.1 | 28.4 | 29.8 |
| West | 2009 | 27.8 | 27.1 | 28.5 |
| West | 2010 | 27 | 26.3 | 27.6 |
| West | 2011 | 27.8 | 27.2 | 28.5 |
| West | 2012 | 26.1 | 25.5 | 26.7 |
| West | 2013 | 26.1 | 25.5 | 26.7 |
| West | 2014 | 24.7 | 24.1 | 25.2 |
| West | 2015 | 24.5 | 23.9 | 25.1 |
| West | 2016 | 23.9 | 23.4 | 24.5 |
| West | 2017 | 25 | 24.4 | 25.6 |
| West | 2018 | 24.1 | 23.5 | 24.6 |
| West | 2019 | 24.1 | 23.5 | 24.6 |
| West | 2020 | 26.7 | 26.2 | 27.3 |
